# Benefit−risk assessment of brensocatib for treatment of non-cystic fibrosis bronchiectasis

**DOI:** 10.1183/23120541.00695-2022

**Published:** 2023-05-02

**Authors:** James D. Chalmers, Mark L. Metersky, Joseph Feliciano, Carlos Fernandez, Ariel Teper, Andrea Maes, Mariam Hassan, Anjan Chatterjee

**Affiliations:** 1 Ninewells Hospital and Medical School, University of Dundee, Dundee, UK; 2 University of Connecticut School of Medicine, Farmington, CT, USA; 3 Insmed Incorporated, Bridgewater, NJ, USA

## Abstract

Bronchiectasis (also referred to as non-cystic fibrosis bronchiectasis [1]) is an inflammatory disease, characterised by permanently dilated bronchi, with chronic cough, sputum production and frequent exacerbations [2, 3]. Increased airway neutrophil elastase (NE) activity is associated with bronchiectasis disease progression and increased risk of pulmonary exacerbations [4, 5]. Brensocatib is an investigational, small-molecule, orally bioavailable, selective, reversible dipeptidyl peptidase 1 inhibitor that blocks activation of neutrophil serine proteases including NE [1, 6, 7].

## Abstract 2

**Brensocatib is a novel anti-inflammatory therapy in development for bronchiectasis treatment. Phase 2 WILLOW trial data demonstrate a low number needed to treat and negative number needed to harm, suggesting a favourable benefit−risk profile.** https://bit.ly/3SbisW3

*To the Editor:*

Bronchiectasis (also referred to as non-cystic fibrosis bronchiectasis [1]) is an inflammatory disease, characterised by permanently dilated bronchi, with chronic cough, sputum production and frequent exacerbations [2, 3]. Increased airway neutrophil elastase (NE) activity is associated with bronchiectasis disease progression and increased risk of pulmonary exacerbations [4, 5]. Brensocatib is an investigational, small-molecule, orally bioavailable, selective, reversible dipeptidyl peptidase 1 inhibitor that blocks activation of neutrophil serine proteases including NE [1, 6, 7]. In the phase 2 randomised, double-blind, placebo-controlled WILLOW study (www.ClinicalTrials.gov identifier NCT03218917 [1]), patients received 10 mg brensocatib (n=82), 25 mg brensocatib (n=87) or placebo (n=87) once daily for 24 weeks [1]. The time to first exacerbation was prolonged with brensocatib compared with placebo (adjusted hazard ratio (HR) 0.58, 95% CI 0.35–0.95 for the 10 mg dose; adjusted HR 0.62, 95% CI 0.38–0.99 for the 25 mg dose) and reductions in sputum NE were observed [1]. The most common serious adverse events (occurring in ≥3% of patients) were infective exacerbation of bronchiectasis (6% for the 10 mg dose; 4% for the 25 mg dose; 11% with placebo) and pneumonia (0% for the 10 mg dose; 4% for the 25 mg dose; 4% with placebo [1]).

To facilitate interpretation of the brensocatib clinical benefit−risk profile, a *post hoc* analysis of the WILLOW study was conducted to calculate the number needed to treat (NNT) and number needed to harm (NNH) for brensocatib compared with placebo in patients with bronchiectasis. NNT and NNH analyses describe the number of patients that would need to be treated for one additional patient *versus* placebo to experience benefit or harm, respectively [8, 9].

The WILLOW study population included adults with computed tomography-confirmed bronchiectasis combined with a relevant clinical history and at least two exacerbations in the previous 12 months. Study details including full inclusion and exclusion criteria, study protocols and information on ethical approval have been published previously [1]. The proportion of patients with pulmonary exacerbations over 24 weeks was used for the NNT analysis and the proportion of patients with serious treatment-emergent adverse events (TEAEs) was used for the NNH analysis. Serious adverse events were defined as any untoward medical occurrence that, at any dose, result in death, are life-threatening, require hospitalisation or prolong existing hospitalisation, result in significant disability/incapacity or are congenital anomalies/birth defects. Since exacerbations were both an efficacy end-point and could be reported as an adverse event, an analysis of NNH was conducted after exclusion of exacerbations reported as serious TEAEs. NNT and NNH were calculated as 1/(*f*_brensocatib_−*f*_placebo_) with 95% CI, where *f*_brensocatib_ is the proportion of brensocatib-treated patients with an exacerbation or serious TEAE, and *f*_placebo_ is the proportion of placebo-treated patients with an exacerbation or serious TEAE. Where the two-sided 95% CI for the risk difference included 0, the 95% CI included infinity. The upper bounds of the 95% CI for all NNH values were infinite (*i.e.* an infinite number of patients would be required to determine the NNH within the 95% CI). An infinite number of people being treated before harm is experienced would be the best possible scenario. Therefore, the worst-case scenario (a positive integer) for the lower bound of the NNH is reported. Negative NNH values suggest a favourable effect of brensocatib treatment on safety parameters *versus* placebo [8].

The brensocatib-treated arms experienced a significantly lower proportion of exacerbations than the placebo-treated arm [1]; the NNTs for exacerbation prevention are presented in table 1. For patients in the brensocatib 10 mg (n=82) arm, the NNT was 6 (95% CI 3–50), due to the lower proportion of patients who experienced exacerbations with brensocatib than with placebo (31.7% *versus* 48.3%, p=0.03 [1]). In the 25 mg (n=87) arm the NNT was 7 (95% CI 3–197) with 33.3% of patients treated with brensocatib experiencing exacerbations (p=0.04) [1]. The NNT in the pooled (n=169) brensocatib treatment group was 6 (95% CI 4–33) with 32.5% experiencing exacerbations with brensocatib.

**TABLE 1:** Numbers needed to treat (NNTs) for exacerbation prevention and numbers needed to harm (NNHs) for serious treatment-emergent adverse events (TEAEs), including and excluding exacerbations

	**Patients**	**Brensocatib**	**Placebo**	**NNT (95% CI)**	**NNH** **(|95% CI|)^#,¶^**
**NNTs for exacerbation prevention (end-point: number with exacerbations^+^)**					
Patients			87		
Brensocatib 10 mg^§^	82	26 (31.7)	42 (48.3)	6 (3−50)	
Brensocatib 25 mg^§^	87	29 (33.3)	42 (48.3)	7 (3−197)	
Brensocatib pooled	169	55 (32.5)	42 (48.3)	6 (4−33)	
**NNHs including exacerbations (end-point: number with serious TEAEs^+^)**					**^¶¶,++^**
Patients			85		
Brensocatib 10 mg ^ƒ^	81	11 (13.6)	19 (22.4)		−11 (>5)
Brensocatib 25 mg^##^	89	10 (11.2)	19 (22.4)		−9 (>5)
Brensocatib pooled	170	21 (12.4)	19 (22.4)		−10 (>5)
**NNHs excluding exacerbations (end-point: number with serious TEAEs^+^ (excluding exacerbations))**					**^++,§§^**
Patients			85		
Brensocatib 10 mg^ƒ^	81	9 (11.1)	11 (12.9)		−55 (>9)
Brensocatib 25 mg^ƒ^	89	8 (9.0)	11 (12.9)		−25 (>8)
Brensocatib pooled	170	17 (10.0)	11 (12.9)		−34 (>9)

Data are presented as n or n (%), unless otherwise stated. ^#^: 95% CI for NNH analyses are reported as absolute values; ^¶^: the two-sided 95% CI of risk difference included 0; therefore, the noncontinuous 95% CI generated indicates that the upper bound of the 95% CI for NNH is infinite (*i.e.* an infinite number of patients would be required to show any harm within the 95% CI); **^+^**: over 24 weeks; ^§^: p≤0.05 *versus* placebo for proportion of patients experiencing exacerbations [1]; ^ƒ^: p*>*0.05 *versus* placebo for proportion of patients experiencing serious TEAEs [1]; ^##^: p≤0.05 *versus* placebo for proportion of patients experiencing serious TEAEs [1]; ^¶¶^: the worst case scenario of lower bound of NNH (including exacerbations) was 5; ^++^: negative NNH values suggest a favourable effect of brensocatib treatment on safety parameters *versus* placebo; **^§§^**: the worst case scenarios of lower bound of NNH (excluding exacerbations) were 9 for brensocatib 10 mg and the brensocatib pooled groups, and 8 for brensocatib 25 mg.

Fewer patients in the brensocatib 10 mg (n=81) arm experienced serious TEAEs over 24 weeks *versus* the placebo (n=85) group (13.6% *versus* 22.4%, p=0.14), and significantly fewer in the brensocatib 25 mg (n=89) arm (11.2%, p=0.049 [1]). The NNH values for the proportion of patients with serious TEAEs, including exacerbations, are presented in table 1. The NNH for the brensocatib 10 mg arm was −11 (|95% CI| >5), and for the brensocatib 25 mg arm the NNH was −9 (|95% CI| >5). The NNH in the pooled (n=170) brensocatib group was −10 (|95% CI| >5), with 12.4% of patients treated with brensocatib experiencing serious TEAEs *versus* 22.4% in the placebo group.

The reduced risk of serious TEAEs was maintained in the results of the NNH analysis excluding exacerbations as a harm. The NNH values for the proportion of patients with serious TEAEs, excluding exacerbations, are presented in table 1. The NNH value excluding exacerbations for the brensocatib 10 mg arm was −55 (|95% CI| >9). The NNH value excluding exacerbations in the 25 mg arm was −25 (|95% CI| >8). p-values for serious TEAEs excluding exacerbations in the 10 mg and 25 mg arms *versus* placebo were 0.19 and 0.79, respectively [1]. In the pooled (n=170) brensocatib group, the NNH value excluding exacerbations was −34 (|95% CI| >9), and 10.0% of patients treated with brensocatib experienced serious TEAEs (excluding exacerbations) *versus* 12.9% of patients receiving placebo.

Exacerbations are critical events in the natural history of bronchiectasis [3]. Frequent exacerbations are associated with a deterioration in quality of life, an increased risk of hospital admission, increased loss of lung function, and mortality [3, 10]. Therefore, an intervention that can prevent patients from experiencing exacerbations over time is of potential clinical importance. As brensocatib is a novel treatment, data on the relative efficacy and safety are important. Clinicians may use NNT and NNH values to better assess the potential benefit−risk profile of an intervention and its possible impact on clinical practice [8, 9]. Here, potential benefit of brensocatib *versus* placebo is suggested by the NNT results, as NNT values of <10 indicate that a treatment has substantial benefit [8].

A potential limitation is that this was a *post hoc* analysis of phase 2 trial results. While there is always a possibility that a phase 3 trial may have different results from its associated phase 2 trial, the findings in this analysis indicate a potential clinical importance, which the ongoing phase 3 ASPEN study (www.ClinicalTrials.gov identifier NCT04594369) aims to substantiate.

Furthermore, in this analysis, the 10 mg brensocatib dose had a lower NNT and more negative NNH than the 25 mg dose, although these results may have been expected of the higher dose. However, it should be noted that this study was not designed to differentiate efficacy by dose.

In conclusion, the analysis discussed here adds to the findings of the WILLOW study. The WILLOW study demonstrated that brensocatib prolonged the time to the first exacerbation and led to a lower risk of exacerbations compared with placebo in patients with bronchiectasis [1]. In the present analysis, the low NNT and negative NNH suggest a potential positive benefit–risk profile of brensocatib. Collectively, these results may indicate that brensocatib could be an important addition to the treatment of patients with bronchiectasis​. The phase 3 ASPEN study is ongoing and aims to confirm these findings.

## References

[1] Chalmers JD, Haworth CS, Metersky ML, et al. Phase 2 trial of the DPP-1 inhibitor brensocatib in bronchiectasis. N Engl J Med 2020; 383: 2127–2137. doi:10.1056/NEJMoa2021713 32897034

[2] Barker AF. Bronchiectasis. N Engl J Med 2002; 346: 1383–1393. doi:10.1056/NEJMra012519 11986413

[3] Chalmers JD, Aliberti S, Filonenko A, et al. Characterization of the “frequent exacerbator phenotype” in bronchiectasis. Am J Respir Crit Care Med 2018; 197: 1410–1420. doi:10.1164/rccm.201711-2202OC 29357265

[4] Keir HR, Shoemark A, Dicker AJ, et al. Neutrophil extracellular traps, disease severity, and antibiotic response in bronchiectasis: an international, observational, multicohort study. Lancet Respir Med 2021; 9: 873–884. doi:10.1016/S2213-2600(20)30504-X 33609487

[5] Chalmers JD, Moffitt KL, Suarez-Cuartin G, et al. Neutrophil elastase activity is associated with exacerbations and lung function decline in bronchiectasis. Am J Respir Crit Care Med 2017; 195: 1384–1393. doi:10.1164/rccm.201605-1027OC 27911604 PMC5443898

[6] Palmér R, Mäenpää J, Jauhiainen A, et al. Dipeptidyl peptidase 1 inhibitor AZD7986 induces a sustained, exposure-dependent reduction in neutrophil elastase activity in healthy subjects. Clin Pharmacol Ther 2018; 104: 1155–1164. doi:10.1002/cpt.1053 29484635 PMC6282495

[7] Doyle K, Lönn H, Käck H, et al. Discovery of second generation reversible covalent DPP1 inhibitors leading to an oxazepane amidoacetonitrile based clinical candidate (AZD7986). J Med Chem 2016; 59: 9457–9472. doi:10.1021/acs.jmedchem.6b01127 27690432

[8] Arnaud A, Suthoff E, Stenson K, et al. Number needed to treat and number needed to harm analysis of the zuranolone phase 2 clinical trial results in major depressive disorder. J Affect Disord 2021; 285: 112–119. doi:10.1016/j.jad.2021.02.027 33640861

[9] Citrome L, Ketter TA. When does a difference make a difference? Interpretation of number needed to treat, number needed to harm, and likelihood to be helped or harmed. Int J Clin Pract 2013; 67: 407–411. doi:10.1111/ijcp.12142 23574101

[10] Brill SE, Patel AR, Singh R, et al. Lung function, symptoms and inflammation during exacerbations of non-cystic fibrosis bronchiectasis: a prospective observational cohort study. Respir Res 2015; 16: 16. doi:10.1186/s12931-015-0167-9 25849856 PMC4324878

